# Visualization of Changes in the Kidneys, Testes, Ovaries, and Uterus of Rats During Plastination, and Their Evaluation by SEM and Histology

**DOI:** 10.3390/vetsci13101093

**Published:** 2026-10-15

**Authors:** Saime Betül Baygeldi, Yeşim Aslan Kanmaz, Ugur Seker, Sadık Yilmaz

**Affiliations:** 1 Department of Anatomy, Faculty of Veterinary Medicine, Fırat University, Elazığ 23119, Türkiye; yesim.aslan@firat.edu.tr (Y.A.K.); syilmaz@firat.edu.tr (S.Y.); 2 Department of Histology and Embryology, Mardin Artuklu University, Mardin 47200, Türkiye; seker.ugur.tr@gmail.com

**Keywords:** silicone plastination, deplastination, rat urogenital organs, tissue shrinkage, organ-specific response, technical limitations, histology, scanning electron microscopy

## Abstract

(1) Background: To address formaldehyde-related health hazards and cadaver scarcity in anatomy education, silicone plastination offers a biosafe alternative, yet organ-specific structural responses at each stage remain inadequately characterized. (2) Methods: This study evaluated sequential microanatomical and ultrastructural changes in rat urogenital organs (kidney, testis, ovary, and uterus; *n* = 20 Sprague–Dawley rats) across four plastination stages (fixation, dehydration, forced impregnation, and gas curing) using light microscopy (H&E, Alcian Blue-PAS) and scanning electron microscopy (SEM). (3) Results: Histological and SEM analyses revealed that tissue architecture was well preserved during initial fixation and dehydration. Conversely, forced impregnation and gas curing induced the most pronounced structural alterations, including tubular shrinkage and false PAS positivity in the testis, parenchymal fragmentation in the ovary, and loss of uterine luminal microvilli. Ultrastructurally, polymer deposition masked glomerular and tubular surface topography in the kidney and testis. Overall, kidney and uterine tissues maintained basic histological recognizability better than gonadal tissues. (4) Conclusions: These findings demonstrate that structural damage peaks during forced impregnation and curing, establishing organ-specific vulnerability and underscoring the necessity of tissue-tailored plastination protocols.

## Simple Summary

Cadaver scarcity and the health risks associated with toxic fixatives such as formaldehyde have driven the adoption of plastination as a safe and durable alternative in veterinary anatomy education. However, how individual organs respond structurally to each step of the plastination process remains incompletely understood. In this study, the urogenital organs (kidney, testis, ovary, and uterus) of rats were evaluated stage-by-stage across the four phases of silicone plastination (fixation, dehydration, forced impregnation, and gas curing) using light microscopy and scanning electron microscopy (SEM). The findings reveal that structural changes and tissue shrinkage peak primarily during forced impregnation and gas curing rather than during initial fixation or dehydration. Furthermore, organ-specific vulnerabilities were identified: while the kidney and uterus maintain their basic histological integrity relatively well, the testis and ovary undergo significant structural alterations and surface degradation during impregnation and curing. These results demonstrate that plastination can preserve tissue architecture for microscopic evaluation while underscoring the need to develop organ-tailored plastination protocols for veterinary education and research.

## 1. Introduction

Preserving anatomical specimens with minimal structural degradation and high morphological fidelity is fundamental to anatomical education and research [1,2]. Techniques for preserving biological tissues for pedagogical, scientific, and museum display purposes date back to antiquity [3,4]. However, conventional embalming methods primarily reliant on liquid fixative solutions often cause undesirable changes in tissue flexibility, natural coloration, and long-term structural integrity [5].

Among alternative preservation techniques, plastination offers an innovative and effective approach for the long-term preservation of very thin sections, enabling detailed microscopic examination while maintaining the structural integrity of sectioned specimens. Rapid advances in imaging techniques have increased the importance of cross-sectional anatomy in recent years. Plastination allows detailed visualization of internal anatomical structures in their normal positions and provides materials for studying and understanding cross-sectional human anatomy [1,6,7]. The technique known as micro-plastination, which was not achievable before the advent of plastination, enables the identification of microscopic features in macroscopic anatomical material and facilitates histological analysis [8].

Silicone plastination relies on a four-stage physicochemical process that permanently preserves biological tissues by replacing intracellular fluids and lipids with a durable polymer matrix. First, primary fixation with neutral buffered formalin arrests autolysis and cross-links tissue proteins, establishing the baseline structural framework. Subsequently, freeze-substitution dehydration with cold acetone replaces cellular water with a volatile organic solvent and solubilizes lipid deposits, leaving an acetone-saturated, highly porous matrix ready for hydrophobic polymer penetration. During forced impregnation under negative pressure in a vacuum chamber, acetone is extracted and vaporized, creating a pressure gradient that draws the silicone resin mixture (S10/S3) deep into interstitial and intracellular spaces. Finally, exposure to a cross-linking agent (S6 gas) in a hermetic curing unit initiates polymerization, transforming the flexible, polymer-infused specimen into a dry, odorless, and structurally resilient material. Elucidating the function and chemical mechanism of each phase provides an essential framework for evaluating how differing tissue architectures interact with the technique across successive processing stages [1,6,7].

Visceral organs exhibit a wide range of histological architecture, parenchymal density, and fluid-to-lipid ratios, suggesting that their structural responses to silicone plastination may vary inherently. Organs with dense muscular or fibrous frameworks, such as the uterus and kidney, differ fundamentally in tissue composition from those with delicate tubular networks, high fluid content, or follicular spaces, such as the testis and ovary [8].

Examining how these distinct architectural frameworks interact with the sequential phases of plastination, including dehydration, forced impregnation, and polymer curing, is essential for evaluating tissue preservation and technical feasibility. Therefore, investigating a representative spectrum of urogenital organs with contrasting structural features provides a comprehensive basis for understanding tissue technique interactions and establishes a rationale for organ-tailored micro-plastination protocols [9,10,11].

In this study, changes in tissue integrity in samples collected at various stages of silicone plastination were assessed using histological parameters and visualized with scanning electron microscopy. To this end, we aim to answer the question: “Do the successive stages of the silicone plastination protocol (fixation, dehydration, forced impregnation, and gas curing) induce ultrastructural and histological changes at comparable levels across urogenital organs with differing histological structures, specifically parenchymal/tubular organs (kidney and testis) versus stromal/follicular/muscular organs (ovary and uterus), or do they result in organ-specific variations?”

## 2. Materials and Methods

In this study, 20 adult Sprague–Dawley rats (10 females and 10 males) obtained from the Fırat University Laboratory Animal Unit were used to harvest kidneys, testes, ovaries, and uteri. The study was designed as a descriptive histological and ultrastructural (SEM) evaluation across sequential plastination stages rather than an inferential quantitative hypothesis-testing model. The sample size was determined in accordance with the 3R principles (Reduction). This ensured a highly representative number of biological replicates to reliably assess organ-specific and stage-dependent microanatomical changes with adequate reproducibility.

### 2.1. Animals and Experimental Design

Urogenital organs were harvested from 20 adult Sprague–Dawley rats (10 males and 10 females) after euthanasia. Rather than categorizing animals by sex, experimental groups were defined by the four sequential stages of silicone plastination to systematically evaluate stage-dependent microanatomical alterations. Biological material was evenly distributed across the four experimental stages, yielding a defined sample size of *n* = 5 kidneys (male), *n* = 5 kidneys (female), *n* = 5 testes, *n* = 5 ovaries, and *n* = 5 uteri per processing stage: Group 1 (Fixation Stage, *n* = 5 per organ type): Samples evaluated immediately after 10% neutral buffered formalin fixation. Group 2 (Dehydration Stage, *n* = 5 per organ type): Samples evaluated after complete grade-increasing acetone dehydration. Group 3 (Forced Impregnation Stage, *n* = 5 per organ type): Samples evaluated after vacuum-assisted silicone (S10/S3) impregnation. Group 4 (Gas Curing Stage, *n* = 5 per organ type): Final plastinated samples evaluated after gas curing (S6 hardener) polymer cross-linking. Representative tissue specimens collected from each stage (*n* = 5 per organ/stage) underwent parallel histological (H&E, PAS) and ultrastructural (SEM) evaluations.

### 2.2. Silicone Plastination Procedures

#### 2.2.1. Sample Preparation and Fixation

Urogenital organs (kidney, testis, ovary, and uterus) were dissected from 20 rats (10 females, 10 males) after euthanasia. Tissues were fixed in 10% neutral buffered formalin (NBF) at room temperature (~20–22 °C) for 48 h at a 10:1 fixative-to-tissue ratio, then rinsed with running tap water for 12 h.

#### 2.2.2. Dehydration and Defatting

Dehydration was performed by sequential baths of pure acetone (99.5%) at −25 °C to replace tissue water via freeze-substitution. Acetone purity was monitored daily with an acetonometer and maintained at ≥98%. Immediately after dehydration, defatting (degreasing) was completed by allowing specimens to stand in the final 100% acetone bath at room temperature (20–22 °C) for 3 days. This step solubilizes and extracts intracellular and interstitial lipid deposits, yielding an acetone-saturated, lipid-depleted porous matrix ready for silicone polymer penetration.

#### 2.2.3. Forced Impregnation

Dehydrated and defatted specimens were submerged in a BIODUR S10/S3 (BIODUR^®^ Products GmbH, Heidelberg, Germany) polymer mixture within a vacuum chamber. Forced impregnation was achieved by gradually increasing the negative pressure to extract and vaporize the volatile acetone, allowing the silicone polymer to progressively replace the solvent within the tissue matrix until complete solvent extraction was achieved.

#### 2.2.4. Gas Curing Stage

Following impregnation, excess polymer was drained from the specimen surfaces, and the specimens were positioned into their desired anatomical shape. The impregnated specimens were then transferred to a sealed curing unit and exposed to BIODUR S6 gas hardener(BIODUR^®^ Products GmbH, Heidelberg, Germany) at room temperature to induce polymer cross-linking and complete hardening. The durations of each silicone plastination stage are presented in Table 1.

### 2.3. Morphometric Measurement and Shrinkage Calculation

All morphometric evaluations were conducted on digital photomicrographs captured under a light microscope using Fiji software (an open-source distribution of ImageJ, NIH, Bethesda, MD, USA; version 2.14.0). Spatial scale calibration was established for each magnification (20× and 40×) using stage micrometer scale bars (Analyze > Set Scale) to convert pixel values into physical units (µm or mm).

For each organ specimen, 5 randomly selected, non-overlapping microscopic fields were measured per section across 3 serial sections per subject, yielding 15 representative measurements per organ per animal. Data are expressed as Mean ± Standard Deviation (Mean ± SD).

Morphometric measurements were performed on calibrated photomicrographs using Image J (version 1.54t;NIH, Bethesda, MD, USA). The percentage of tissue shrinkage across sequential plastination stages was calculated relative to the initial baseline value using the following formula:

Shrinkage (%) = [(D_baseline − D_stage)/D_baseline] × 100

where D_baseline is the initial mean dimensional measurement obtained immediately after 10% neutral buffered formalin fixation (Group 1: Baseline Control), and D stage is the corresponding measurement recorded after each sequential processing stage (Group 2: Dehydration/Defatting, Group 3: Forced Impregnation, or Group 4: Gas Curing). All data are presented as descriptive statistics in the form of Mean ± Standard Deviation (Mean ± SD).

### 2.4. Procedure for Histological Tissue Tracking

#### 2.4.1. Tissue Tracking Protocol

For the early processing stages (fixation and dehydration controls), tissue specimens were washed under running tap water for 12 h, dehydrated through a graded ethanol series (70–100%), cleared in xylene, and embedded in paraffin. For the advanced plastinated stages (forced impregnation and gas curing), specimens were soaked in methanol for 24 h, deplastinated in a freshly prepared 5% sodium methoxide solution until adequately softened, cleared in xylene for 20 min, and then embedded in paraffin. Serial 5-µm-thick sections from all embedded blocks were mounted on positively charged slides and stained with Hematoxylin and Eosin (H&E) for routine morphological examination, and with Alcian Blue–PAS to evaluate testicular and glomerular basement membrane integrity.

#### 2.4.2. Hematoxylin-Eosin and PAS Staining Protocol

For the baseline control group (Group 1: Fixation Stage), formalin-fixed, non-plastinated tissues were washed under running tap water for 12 h, dehydrated through a graded ethanol series (70–100%), cleared in xylene, and embedded in paraffin using routine histological techniques.

Paraffin-embedded sections (5 µm) of urogenital tissues (testis, epididymis, kidney, ovary, and uterus) from all processing stages, along with fresh control tissues, were deparaffinized in xylene, rehydrated through graded alcohols, and brought to distilled water. For routine morphological evaluation, sections were stained with H&E. Serial 5-µm-thick sections mounted on positively charged slides were stained with Hematoxylin and Eosin (H&E) for routine morphology, and with a commercial Alcian Blue–PAS (pH 2.5) staining kit [PatoLab, İstanbul, Türkiye. Katalog no: Kit PLKit6-150] according to the manufacturer’s instructions to evaluate testicular and glomerular basement membrane integrity. To assess basement membrane integrity and carbohydrate distribution, parallel sections were stained with Alcian Blue-PAS using a ready-to-use kit per the manufacturer’s instructions. True PAS reactivity was defined as sharp, linear staining strictly confined to basement membranes, whereas diffuse background reactivity from tissue condensation was considered nonspecific background staining. All stained sections were cleared in xylene, mounted with Entellan, and examined under a light microscope.

#### 2.4.3. Deplastination Protocol

For the deplastination of silicone-impregnated tissues prior to paraffin embedding, a 5% (*w*/*v*) sodium methoxide solution was prepared by dissolving 5 g of sodium methoxide powder (CH_3_ONa) in 100 mL of absolute methanol. Plastinated urogenital tissue specimens were completely submerged in this freshly prepared solution at a volume-to-tissue ratio of approximately 15:1 (~25 mL of solution per tissue specimen). Tissues were incubated in the solution at room temperature for 24–48 h, with periodic manual checks using a fine needle to confirm adequate tissue softening before proceeding to xylene clearing and paraffin embedding.

#### 2.4.4. Procedure for the Scanning Electron Microscope (SEM)

Ultrastructural surface evaluation of urogenital tissue samples (kidney, testis, ovary, and uterus) across all four experimental stages was conducted using a scanning electron microscope (FEI Quanta FEG250, Thermo Fisher Scientific, Eindhoven, The Netherlands) at the Aksaray University Central Research Laboratory.

Sample Preparation & Fixation: For scanning electron microscopy (SEM) evaluation, representative tissue specimens were obtained sequentially from each of the four experimental processing groups (Group 1: Formalin-fixed baseline controls; Group 2: Acetone-dehydrated/defatted; Group 3: Forced impregnated; Group 4: Gas cured). Specimens were fixed in 2.5% glutaraldehyde in 0.1 M phosphate-buffered saline (PBS, pH 7.4) at 4 °C for 24 h. Following fixation, samples were washed three times in 0.1 M PBS (10 min each). Specimens were then post-fixed in 2.5% glutaraldehyde, dehydrated through graded ethanol, critical-point dried, sputter-coated with gold-palladium, and examined under a scanning electron microscope. Representative tissue specimens from each stage group were trimmed into standardized small blocks.

Dehydration & Drying: Specimens were dehydrated through a graded ethanol series (30%, 50%, 70%, 80%, 90%, 95%, and 100% *v*/*v*; 15 min per bath). Dehydrated samples were air-dried in a vacuum desiccator.

Conductive Coating: Dried specimens were mounted onto aluminum stubs using double-sided conductive carbon tape and sputter-coated with a gold (Au) conductive layer approximately 10–15 nm thick using a sputter coater (Leica Microsystems GmbH, Wetzlar, Germany)operated for 120 s at 20 mA.

Imaging Parameters: Micrographs were captured using a Secondary Electron (SE) detector (FEI Company, Eindhoven, The Netherlands) at an accelerating voltage of 15 kV and a working distance (WD) of 10–13 mm. Qualitative ultrastructural evaluation was performed at magnifications of 100× to 5000×. To ensure representative observations and reproducibility, at least 5 randomly selected micrographs were examined per tissue specimen across [*n* = 5] independent biological samples in each processing group.

## 3. Results

### 3.1. Results of Silicone Plastination

**Figure 1:**
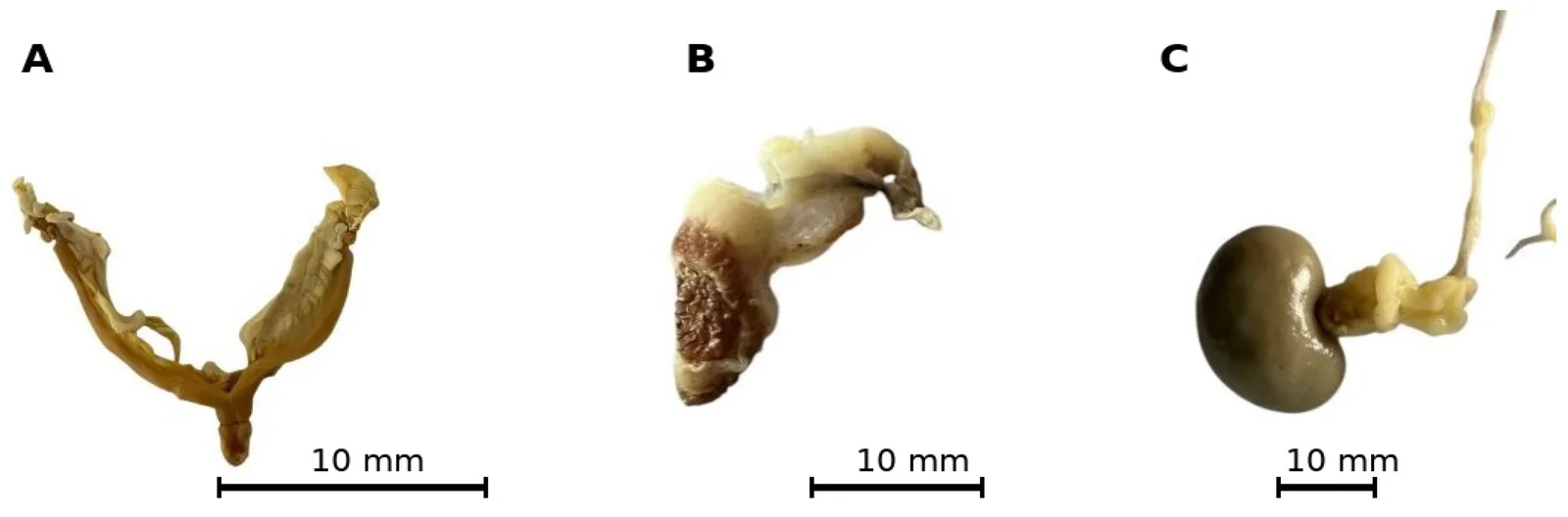
Silicone plastinated urogenital organs of the rat: (**A**) uterus and ovary, (**B**) testis, and (**C**) kidney. Scale bars = 10 mm.

The durations of the various stages of the silicone plastination process performed on rat urogenital organs are shown in Table 1. Macroscopic evaluation revealed that room-temperature silicone plastinates retained their characteristic organ topographies and overall structural dimensions, with identifiable external anatomical boundaries across all urogenital samples, along with stage-dependent dimensional reductions (Figure 1).

### 3.2. Quantitative Stage-Dependent Tissue Shrinkage Results

Morphometric evaluation across the four sequential silicone plastination stages revealed distinct, stage-dependent and organ-specific variations in structural dimensions (Table 2). Initial acetone dehydration (Group 2) produced only minor, non-significant dimensional reductions across all urogenital specimens relative to the post-fixation baseline. In contrast, the most pronounced dimensional shrinkage occurred during forced impregnation (Group 3) and final gas curing (Group 4). Gonadal tissues were most susceptible to volume loss, with testicular seminiferous tubule diameter decreasing by a cumulative 27.6 ± 3.1% and ovarian parenchymal surface area by 24.2 ± 3.5% at the final gas-cured stage. Conversely, organs supported by dense capsular or muscular frameworks demonstrated superior dimensional stability, presenting organ-specific total cumulative shrinkage rates of 11.2 ± 1.8% in renal glomerular cross-sectional area and 13.5 ± 2.1% in uterine luminal diameter, respectively (Table 2).

### 3.3. Findings from Hematoxylin and Eosin-Stained Specimens

**Figure 2:**
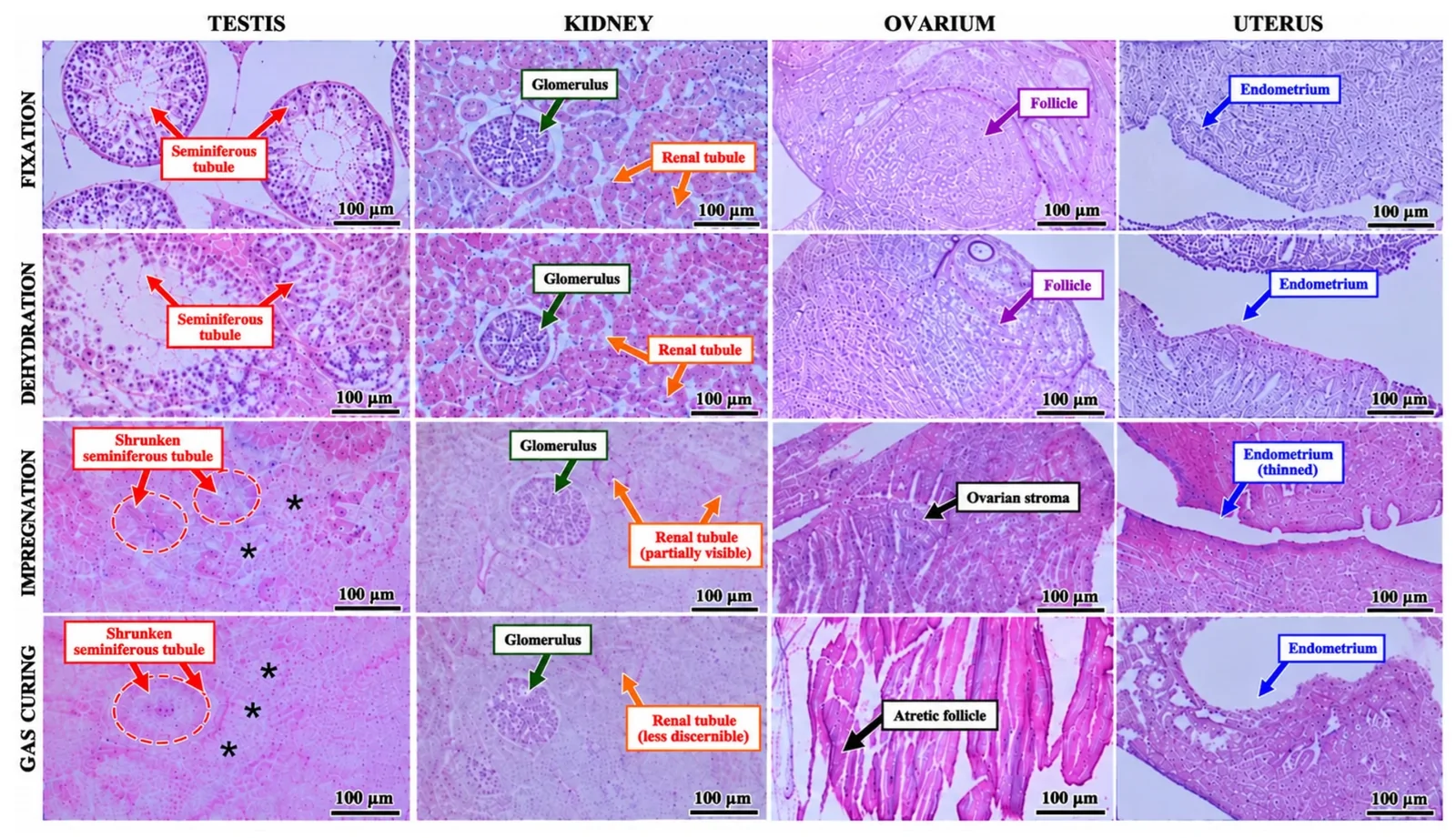
Representative histological micrographs of testicular, renal, ovarian, and uterine tissues following fixation, dehydration, forced impregnation, and gas curing. Testis: During fixation and dehydration, seminiferous tubules including spermatogonia, remained distinguishable (red arrows). Following forced impregnation and gas curing, apparent shrinkage and distortion of seminiferous tubules were observed (red arrows and dashed circles). Asterisks indicate intertubular regions exhibiting morphological alterations. Kidney: Glomeruli (green arrows) and renal tubules (orange arrows) were clearly distinguishable following fixation and dehydration. After forced impregnation and gas curing, glomeruli remained identifiable, whereas renal tubular structures became progressively less discernible (orange arrows). Ovary: Ovarian follicles (purple arrows) were identifiable in tissues subjected to fixation and dehydration. Following forced impregnation, alterations in ovarian tissue architecture were observed, with ovarian stromal regions indicated by black arrows. After gas curing, marked structural disruption was evident, with a region interpreted as an atretic follicle indicated by a black arrow. Uterus: The endometrium (blue arrows) was distinguishable following fixation and dehydration. After forced impregnation, apparent thinning and morphological distortion of the endometrium were observed (blue arrow), while the myometrium remained identifiable. Following gas curing, the endometrial and myometrial regions remained partially distinguishable despite alterations in tissue architecture. Scale bars = 100 µm.

It was observed that the testicles, which had undergone fixation and dehydration, exhibited normal and similar morphological characteristics. In the testicular tissues subjected to forced impregnation and gas curing, seminiferous tubules exhibited noticeable structural shrinkage, accompanied by a reduction in intertubular luminal spaces (asterisk). Nevertheless, spermatogonia, germ cell nuclei across all spermatogenic stages, and interstitial Leydig cells remained clearly identifiable within the testicular architecture (Figure 2).

In kidney sections, it was observed that samples subjected to fixation and dehydration retained normal renal histological features, whereas in samples treated with impregnation and gas clearing, glomeruli were visible, but renal tubular structures were only partially discernible (Figure 2).

Upon examination of the ovarian sections, normal ovarian morphology was observed in the fixation and dehydration groups. In tissue samples subjected to impregnation and gas curing, sectioning was difficult, and fragmentation of the ovarian parenchyma was observed. Nevertheless, the ovarian cortex was distinguishable, and large follicles and atretic follicular remnants were detectable.

Uterine sections from the fixation and dehydration groups demonstrated well-defined endometrium, myometrium, and perimetrium layers, with the uterine lumen lined by an intact epithelium.

It was concluded that no structural damage occurred to the overall morphology of the impregnated uterine tissue sections, and that light microscopic examination and morphological evaluation could be clearly performed after deplastination. In these sections, the uterine glands exhibited normal morphology. In uterine sections treated with gas curing, routine histological examination revealed that the uterine histological layers could be distinguished; however, in uterine tissues subjected to deplastination following gas curing, methodological flaws such as shedding and the formation of thick sections in some areas were identified due to the inability to completely remove the plastination material, though these could be improved through optimization.

Optimized plastination protocols may enable uterine tissue to exhibit morphological characteristics similar to those of baseline control tissue samples after deplastination (Figure 2).

In general, no disruption of tissue integrity was observed in any of the organs during the fixation and dehydration stages of silicone plastination. However, alterations were observed in organs other than the kidney and uterus during the demanding impregnation and gas-curing stages. In contrast, the general tissue architecture and normal histological characteristics of the kidney and uterus specimens were preserved throughout all stages of silicone plastination, although some partial artifacts were present.

### 3.4. Morphological Characteristics of PAS-Stained Testis and Kidney Sections

**Figure 3:**
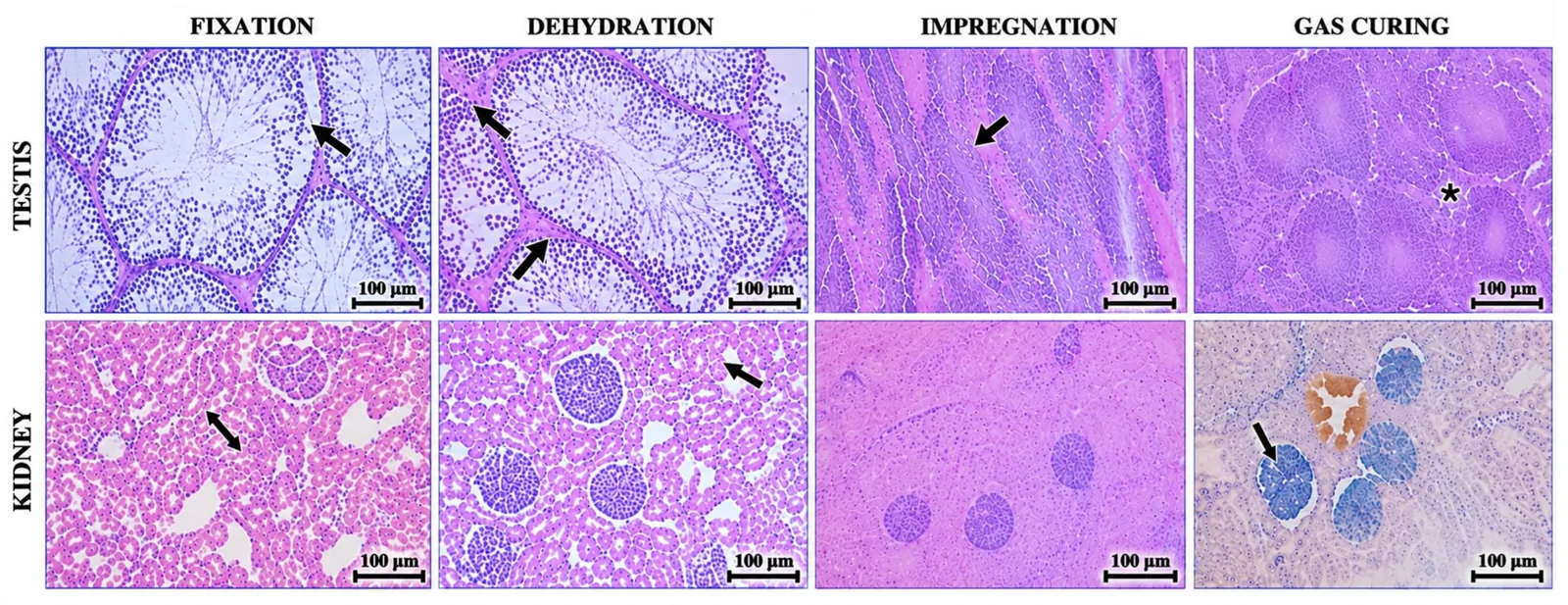
Tissue sections stained with Alcian PAS and treated with fixation, dehydration, impregnation, and gas diffusion. Testicular (arrow) basement membranes; indistinguishable basement membranes (thick arrow) and shedding in the intertubular space (asterisk) in testicular tissues subjected to impregnation and gas flushing. Despite partial PAS positivity in the kidney subjected to impregnation, the glomeruli in the kidney subjected to gas flushing exhibit sharp PAS negativity. Staining: Alcyan PAS Stain. Scale bar: 50 µm.

PAS staining was performed on testis and kidney sections obtained following the protocols to examine the basal membrane and glycogen deposition. In the testis and kidney sections subjected to fixation and dehydration, PAS positivity was observed as expected. In the testis sections, the basal membrane structure of the seminiferous tubules exhibited PAS positivity, and the glomerular basal membrane structure also showed a positive PAS reaction as expected. In the groups treated with impregnation and gas curing, nonspecific PAS reactivity was observed covering diffuse areas in the intertubular region of the testicular sections. Upon examination of the kidney sections, atypical background staining associated with tissue condensation was detected across the renal tubular system. However, in sections treated with gas curing, despite faint background reactivity in the tubular system, glomerular structures remained unresponsive to Schiff’s reagent, reacting exclusively with Alcian Blue. It was concluded that PAS positivity did not occur because the glomerular structures were stained only with Alcian Blue and were unaffected by periodic acid and Schiff’s reagent (Figure 3).

In our PAS-stained evaluations, true basement membrane reactivity was readily distinguished from processing-induced false PAS positivity by localization, sharpness, and chromatic contrast. Fixed and dehydrated tissues showed sharp, linear basement membrane positivity, whereas forced impregnation and gas curing caused intense tissue condensation and siloxane polymer masking. This produced diffuse intertubular false positivity in testicular tissue and stromal condensation in kidney sections. The absence of PAS reactivity with exclusive Alcian Blue staining in gas-cured glomeruli further confirms that advanced plastination stages obscure native carbohydrate epitopes, underscoring the need to compare plastinated specimens with unprocessed stage controls.

### 3.5. Scanning Electron Microscopy Findings

**Figure 4:**
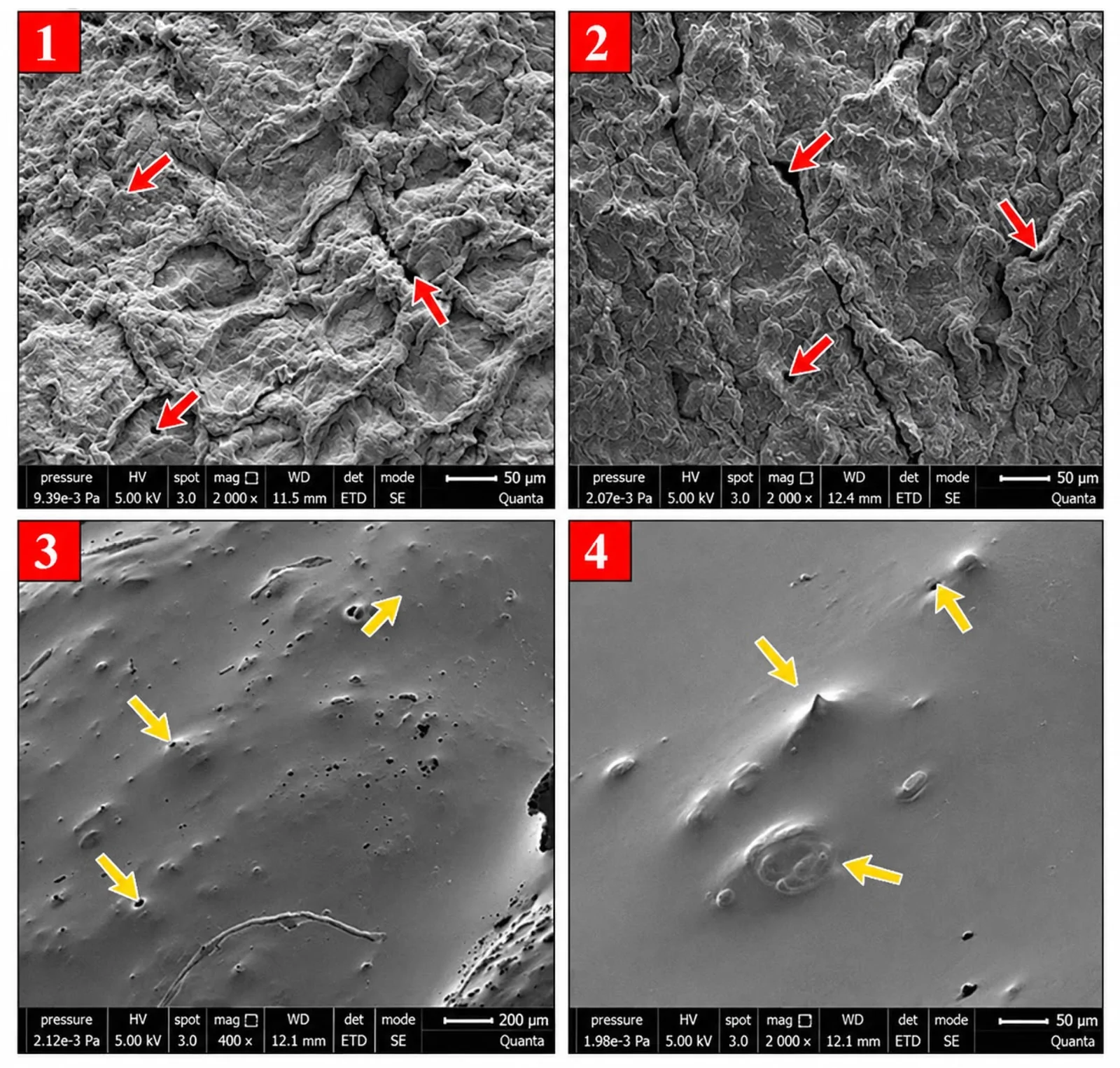
Scanning electron micrographs illustrating morphological variations in ovarian surface tissue architecture. (**1**) Fixation: Rough and irregular surface morphology characterized by prominent folds, ridges, and heterogeneous structural organization (red arrows). (**2**) Dehydration: Highly irregular and wrinkled surface exhibiting pronounced structural discontinuities and localized fissures (red arrows). (**3**) Forced impregnation: Relatively smooth and homogeneous surface with scattered small depressions, pores, and localized surface irregularities (yellow arrows). (**4**) Gas curing: Predominantly smooth and compact surface morphology with occasional protrusions and shallow depressions (yellow arrows). Magnifications: (**1**) 2000×, (**2**) 2000×, (**3**) 400×, and (**4**) 2000×. Scale bars: (**1**,**2**,**4**) 50 µm; (**3**) 200 µm.

**Figure 5:**
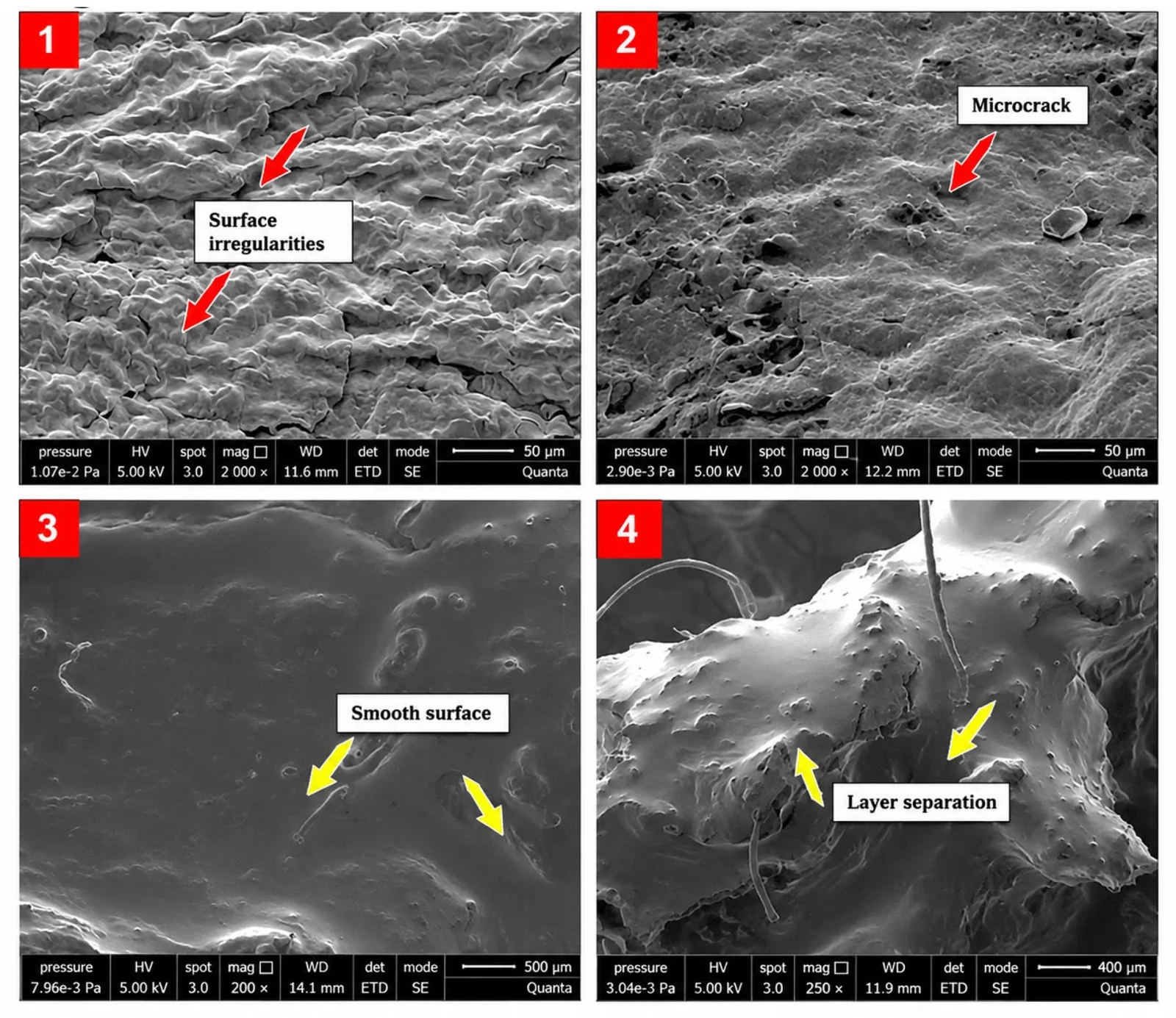
Scanning electron micrographs illustrating morphological variations in uterine tissue surface architecture. (**1**) Fixation: Rough and irregular surface morphology characterized by prominent ridges, folds, and heterogeneous surface organization (red arrows). (**2**) Dehydration: Irregular and heterogeneous surface exhibiting localized microcracks, small depressions, and structural discontinuities (red arrow). (**3**) Forced impregnation: Relatively smooth and homogeneous surface with occasional shallow depressions and minor surface irregularities (yellow arrows). (**4**) Gas curing: Irregular surface architecture characterized by pronounced folds, localized protrusions, and areas of apparent layer separation (yellow arrows). Magnifications: (**1**) 2000×, (**2**) 2000×, (**3**) 200×, and (**4**) 250×. Scale bars: (**1**,**2**) 50 µm, (**3**) 500 µm, and (**4**) 400 µm.

**Figure 6:**
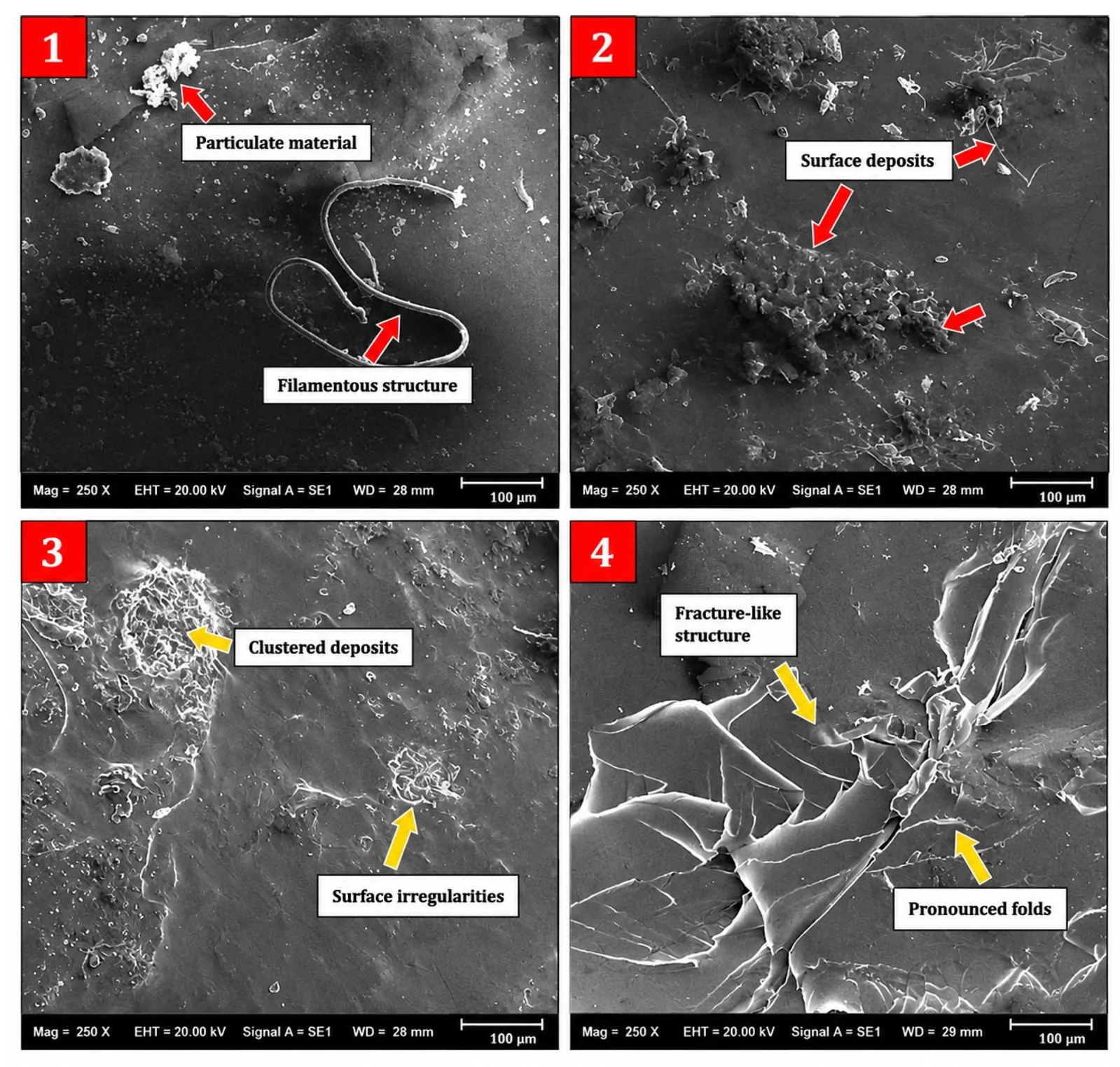
Scanning electron micrographs illustrating variations in kidney tissue surface morphology and microstructural characteristics. (**1**) Fixation: A relatively smooth surface with scattered particulate material (upper red arrow) and an elongated filamentous structure (lower red arrow). (**2**) Dehydration: A heterogeneous surface with irregularly distributed particulate deposits and localized accumulations of surface material (red arrows). (**3**) Forced impregnation: Surface morphology marked by clustered deposits and localized structural irregularities (yellow arrows). (**4**) Gas curing: Pronounced surface disruption with extensive folds, fracture-like structures, and apparent separation of superficial layers (yellow arrows). All micrographs were obtained at 250× magnification. Scale bars represent 100 µm.

**Figure 7:**
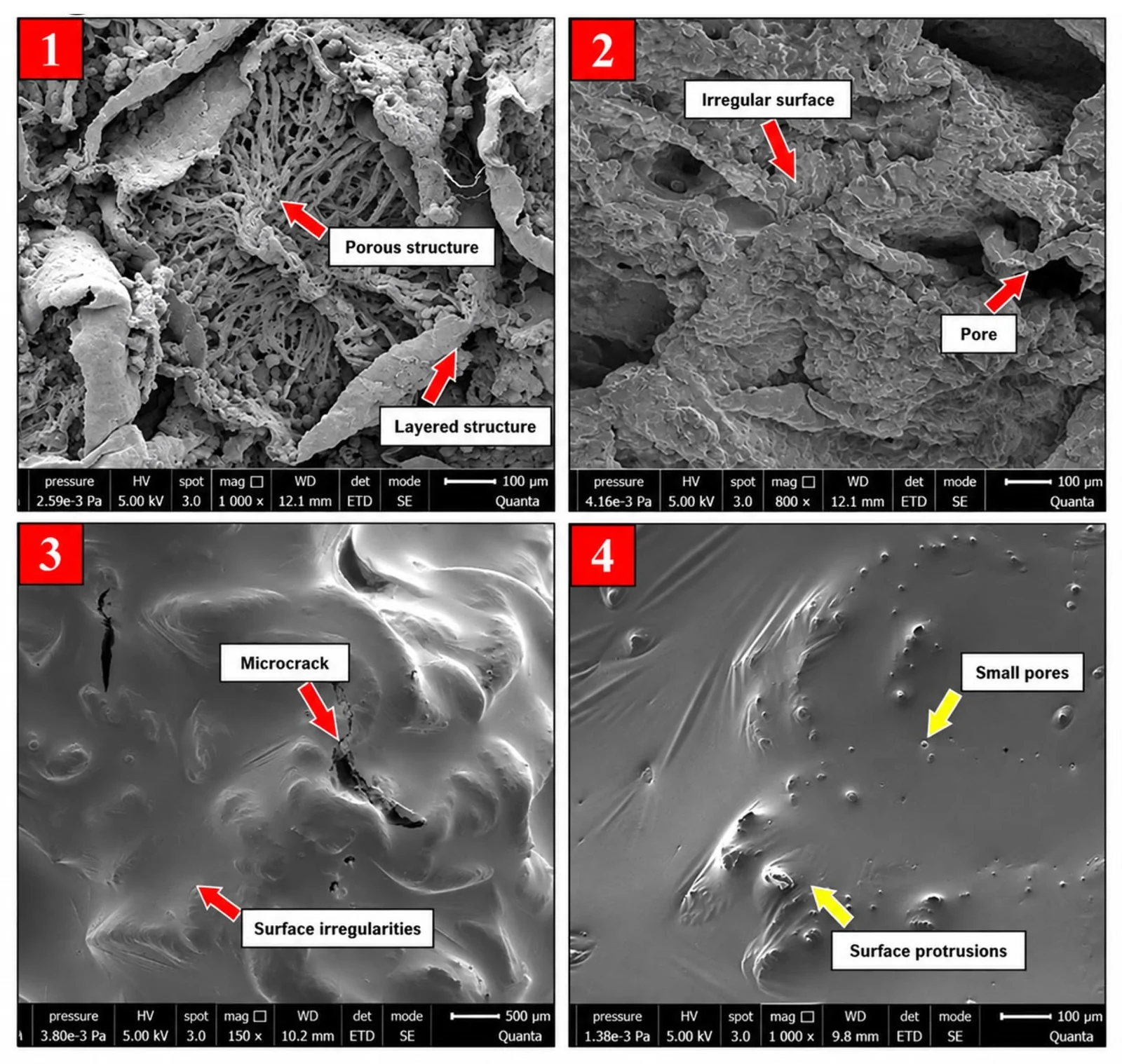
Scanning electron micrographs illustrating variations in testis tissue microarchitecture and surface morphology. (**1**) Fixation: Highly porous and interconnected network like structure with distinguishable layered components and irregularly arranged tissue elements (red arrows). (**2**) Dehydration: Heterogeneous and irregular surface morphology characterized by compacted tissue components, localized depressions, and prominent pores (red arrows). (**3**) Forced İmpregnation: Relatively smooth surface exhibiting localized microcracks, pronounced surface undulations, and structural irregularities (red arrows). (**4**) Gas curing: Predominantly smooth and compact surface with scattered small pores, localized protrusions, and subtle surface folds (yellow arrows). Magnifications: (**1**) 1000×, (**2**) 800×, (**3**) 150×, and (**4**) 1000×. Scale bars: (**1**,**2**,**4**) 100 µm; (**3**) 500 µm.

Images of the scanning electron microscopy findings, categorized into 4 groups (Group 1: fixation; Group 2: dehydration; Group 3: forced impregnation; Group 4: gas curing) (Figure 4, Figure 5, Figure 6 and Figure 7), are presented below.

The images for the fixation and dehydration groups in the ovaries were normal. In the impregnation and gas curing groups, however, erosion of the surface epithelium was observed (Figure 4).

The endometrial layer of the uterus contained numerous microvilli. The endometrium contains openings that lead to tubular uterine glands beneath the epithelial surface. In Groups 3 and 4, the microvilli had disappeared, and the intervening spaces had become significantly more numerous (Figure 5).

In the fixation and dehydration groups, glomeruli and adjacent tubules in the renal cortex were clearly visible. Podocyte branching processes were observed wrapping around the glomerular capillaries, and the inner surface of Bowman’s capsule displayed long, solitary cilia extending from the flattened parietal epithelial cells. However, in Groups 3 and 4, glomerular surface details were markedly distorted by silicone polymer accumulation and ultrastructural shrinkage (Figure 6).

In Groups 3 and 4, silicone polymer deposition heavily coated the renal cortical surface, obscuring the underlying surface topography of glomeruli and podocyte processes. This surface masking effect observed in SEM correlated with light microscopy findings, where Bowman’s space appeared narrowed and tubular boundaries condensed (Figure 6).

Scanning electron microscopy of Group 1 testicular tissue revealed well-preserved seminiferous tubules (ST) bounded by peritubular myoid cells (M) and intervening interstitial spaces (IS) (Figure 7). Distinct germ cell layers were clearly identifiable within the seminiferous tubules, and the tubular lumens were filled with flagellated spermatozoa (Figure 7).

In Group 2 (dehydration), SEM findings were nearly identical to those of the baseline control group, with tubular lumens densely filled with long-flagellated spermatozoa and a mild reduction in interstitial spaces. In Groups 3 (forced impregnation) and 4 (gas curing), silicone polymer deposition coated the surface lining, obscuring most seminiferous tubules.

In conclusion, scanning electron microscopy revealed that urogenital tissue specimens (ovarian, uterine, renal and testicular) generally retained their characteristic morphological features during fixation and dehydration. Conversely, the demanding forced impregnation and gas curing stages introduced both localized and widespread disruptions to tissue ultrastructural integrity.

## 4. Discussion

Traditional systematic anatomy education often falls short in preparing health science students for advanced clinical applications and radiological image interpretation, both of which fundamentally require a clear grasp of cross-sectional relationships [12,13,14,15]. Furthermore, conventional embalmed specimens preserved in liquid solutions deteriorate rapidly over time, and thin cross-sections are particularly prone to structural separation and reduced clarity. In agreement with Siddiquey et al. [16], silicone plastination offers significant advantages for preserving cross-sectional anatomical structures, yielding durable, dry, and macroscopically resilient pedagogical materials that facilitate 3D spatial understanding of complex organ systems. However, a rigorous evaluation of the technique requires recognizing its inherent limitations: while gross anatomical relationships are largely retained, forced impregnation and gas-curing stages introduce notable stage-dependent microanatomical shrinkage, surface disruptions, and ultrastructural alterations across delicate tissue layers. Consequently, while silicone plastinated specimens serve as safe and long-lasting educational tools, their efficacy for high-resolution micro-level evaluations must be interpreted with caution, taking these technique-induced structural trade-offs into account.

Although sectional plastination is conventionally performed with epoxy or polyester resins, silicone plastination (S10) remains the most versatile method for whole cadavers, intact organs, and sectioned specimens [6,17]. Consistent with Pendovski et al. [18] and Steinke et al. [7], who demonstrated that the S10 technique could produce structural organ sections at least 2–3 mm thick, our findings confirm that silicone plastination can be successfully adapted for urogenital organ sectioning, yielding highly detailed and durable specimens. Furthermore, the operational parameters of forced impregnation significantly influence both processing efficiency and specimen quality. While classical protocols often use low-temperature impregnation, forced impregnation at room temperature accelerates silicone penetration, shortens processing time, and yields non-sticky specimens [19,20]. By combining low-temperature freeze-substitution dehydration with room-temperature forced impregnation, as recommended by Sagoo [12], we achieved efficient polymer-solvent exchange and preserved structural integrity across the rat urogenital specimens.

Tissue shrinkage is an inherent physical phenomenon driven by fluid displacement and polymer infiltration during plastination [21]. Our quantitative morphometric findings demonstrate distinct, stage-dependent, and tissue-specific shrinkage dynamics across rat urogenital organs. Acetone dehydration produced minimal dimensional reduction across all organ types, indicating that freeze-substitution preserves tissue volume relatively well during initial solvent replacement. In contrast, forced impregnation and final gas curing accounted for the vast majority of cumulative shrinkage. This stage-dependent progression reflects negative pressure gradients created during forced vacuum extraction of acetone, followed by siloxane polymer cross-linking and surface drying during gas curing [11,21]. Furthermore, the degree of shrinkage varied significantly with organ histology and structural framework: delicate, fluid-rich gonadal tissues, specifically testicular seminiferous tubules (27.6 ± 3.1% reduction) and ovarian parenchyma (24.2 ± 3.5% loss), exhibited the greatest vulnerability to volume loss under vacuum and polymerization stress. Conversely, organs supported by dense fibrous capsules or thick smooth muscle layers, such as renal glomeruli (11.2 ± 1.8%) and uterine tissue (13.5 ± 2.1%), demonstrated superior mechanical stability and resistance to compaction. These quantitative results confirm that organ composition and parenchymal density fundamentally govern tissue response throughout sequential plastination stages [11,21].

Understanding how organ-specific architecture dictates these physical volume changes provides an essential foundation for accurately interpreting microscopic integrity. In this context, the advent of micro-plastination, evolving from the broader ‘histological plastination’ concept, has significantly broadened the scope of morphological research by enabling detailed microscopic and histological evaluations on macroscopic tissue sections thinner than 150 µm [8,10,22,23].

As highlighted in our previous study [9] and by Da Silva et al. [24], this technique provides an invaluable research tool for examining micro-anatomical architecture in intact biological specimens. In terms of tissue preservation, Manjunatha et al. [25] and Sawad et al. [26] reported that uncured silicone-plastinated porcine and camel organs preserved histological structures under light microscopy without evident shrinkage. Conversely, López-Albors et al. [27] demonstrated that the polymer curing process fundamentally influences tissue preservation following deplastination [28]. Consistent with López-Albors et al. [27], our study revealed that while initial fixation and dehydration maintained general tissue morphology, the subsequent mandatory forced impregnation and gas curing stages introduced observable structural alterations and shrinkage artifacts.

Although tissue deplastination remains a challenging procedure, accumulating literature demonstrates its feasibility for retrospective histological evaluation. Pioneering studies by Walker et al. [28] and Grondin et al. [29], along with more recent investigations by Rahul et al. [21], established that a 5% sodium methoxide solution in methanol effectively breaks down siloxane polymer cross-links, enabling histological processing. We agree with these foundational studies on the imperative for protocol standardization. However, relying on a fixed 48-h incubation period as a definitive endpoint—as originally described by Grondin et al. [29] is inherently unreliable. The exact duration required for complete depolymerization varies with specimen volume, the ratio of plastinate to deplastination solution, tissue parenchymal density, and the progressive strengthening of polymer cross-linking over time. Therefore, physical monitoring parameters, such as regular manual verification of tissue softness, provide a more accurate criterion for determining full deplastination.

Furthermore, while sodium methoxide yields high-quality depolymerization in small histological specimens, alternative chemical agents, including methylbenzene, methylene, and acetone dichloride, have been evaluated by Ripani et al. [30] and Baygeldi et al. [9] for larger organ specimens. Although Ramos et al. [31] reported that non-deplastinated kidney specimens retain certain tubular and corpuscular landmarks under direct microscopic observation, direct microtome sectioning of fully cured silicone plastinated tissues is practically infeasible without prior polymer extraction.

Our findings confirm that deplastination yields good histological results, underscoring its crucial role as a methodological prerequisite that enables subsequent microscopic and histological examination of plastinated organs. Previous investigations [9,21,26] provide strong evidence that solid organs, such as the liver and kidney, remain visible under light microscopy after deplastination. Consistent with these findings, our routine histological examinations revealed that the morphological and cellular structures of the rat urogenital tissues were partially preserved after deplastination, allowing microscopic evaluation.

Regarding stage-dependent dimensional changes, Medina et al. [11] reported that plastination chemicals induce progressive shrinkage across processing stages, yet overall cellular architecture and tissue details are largely preserved. Correspondingly, in our study, tissue shrinkage occurred primarily during the demanding forced impregnation and gas-curing stages, whereas initial fixation and dehydration preserved general organ morphology. Although structural condensation was observed during polymer replacement, tissue integrity was maintained, particularly in dense parenchymal frameworks.

Furthermore, numerous researchers [6,32,33,34] have adapted the histological plastination approach, demonstrating that resin-embedded specimens preserve cellular details comparable to paraffin-embedded tissues and serve as stable, durable models for optical visualization and three-dimensional digital reconstruction.

In our study, light and scanning electron microscopy (SEM) evaluations across sequential processing stages showed clear, artifact-free ultrastructural images during fixation and dehydration. However, variable artifacts and polymer deposition were observed during forced impregnation and gas curing, depending on tissue density and organ type.

Overall, silicone plastination yields specimens that closely resemble natural, unprocessed tissue in gross morphology. Although shrinkage artifacts arise during polymer curing, these dimensional changes remain within an acceptable range compared with those routinely induced by standard histological tissue processing. To further minimize shrinkage in future applications, optimizing fixative concentrations, modifying dehydration protocols, or gradually infusing silicone oils of increasing viscosity may be beneficial. Future studies should extend this methodology to additional organ systems and incorporate advanced diagnostic tools, such as immunohistochemistry and magnetic resonance imaging (MRI), to evaluate the micro-anatomical and molecular effects of plastination in greater detail.

It is worth emphasizing that conventional histological processing itself inherently induces noticeable shrinkage artifacts, often compromising the three-dimensional architecture of biological tissues. Consequently, the dimensional alterations associated with silicone plastination remain well within acceptable limits compared with standard histological procedures. To further mitigate tissue shrinkage in future applications, lowering formalin fixation concentrations or exploring alternative fixatives that prevent autolysis without causing excessive tissue hardening may prove beneficial. Additionally, optimizing forced impregnation through a stepwise infusion of silicone polymers with increasing viscosity can further preserve delicate parenchymal structures. Overall, silicone-plastinated specimens maintain spatial anatomical relationships effectively, offering a highly practical and reliable alternative to live or formalin-fixed tissues and conventional histological sections, particularly for three-dimensional optical imaging and micro-anatomical visualization.

## 5. Conclusions

In conclusion, this study demonstrates that although silicone plastination generally preserves the gross macro-anatomical architecture of rat urogenital organs, structural shrinkage and ultrastructural alterations are strongly stage-dependent, occurring primarily during forced impregnation and gas curing rather than during initial fixation or dehydration. Crucially, our step-by-step monitoring shows that identical plastination protocols do not produce uniform effects across organ types. Parenchymal organs with dense frameworks, such as the kidney and uterus, maintain histological integrity throughout all plastination stages. In contrast, delicate gonadal tissues (testis and ovary) exhibit significant structural vulnerabilities, including parenchymal fragmentation, tubular disruption, and surface erosion during advanced curing phases.

Furthermore, the parallel integration of ultrastructural (SEM) and histological (H&E, PAS) data provides critical methodological depth; for example, while internal glomerular architecture remains recognizable under light microscopy, SEM surface analysis shows that advanced impregnation and curing induce polymer masking and surface shrinkage. Overall, these findings highlight that standard plastination protocols cannot be universally applied to every tissue type without organ-specific optimization.

When gas curing and impregnation parameters are appropriately tailored, silicone plastination offers a highly effective, safe, and durable alternative for both cross-sectional anatomical education and retrospective histomorphological research.

## Figures and Tables

**Table 1:** Summary of stage-specific protocols and timeframes for silicone plastination of rat urogenital organs.

Plastination Stage	Reagents & Chemicals Used	Experimental Conditions & Criteria	Duration (Days)
Specimen Preparation & Dissection	Fresh urogenital tissue (kidney, testis, ovary, uterus)	Post-euthanasia dissection, removal of excess connective/adipose tissue, and sample trimming	2 days
Dehydration	Pure Acetone (≥99.5% purity, 4 sequential baths)	Freeze-substitution at −25 °C; 10:1 volume-to-tissue ratio; bath changes guided by hydrometer readings until purity exceeds 95%	21 days
Degreasing (Defatting)	Pure Acetone	Incubation in acetone at room temperature (~20–22 °C) to solubilize remaining tissue lipids	3 days
Forced Impregnation	BIODUR S10/S3 Polymer Mixture (100:1 ratio)	Vacuum-assisted solvent extraction at room temperature: 6 days of active vacuum + 1 day of static rest + 4 days of silicone drainage	11 days
Gas Curing (Polymerization)	BIODUR S6 Cross-linking Agent	Hermetic curing chamber exposure for final cross-linking and surface hardening	4 days
Total Process Duration	—	Complete execution of the silicone plastination stages for rat urogenital specimens	41 days

**Table 2:** Dimensional measurements and percentage shrinkage across sequential silicone plastination stages in rat urogenital organs. (Data presented as Mean ± Standard Deviation [Mean ± SD], *n* = 5 biological specimens per organ/stage).

Organ/Morphometric Parameter	Group 1 (Fixation)	Group 2 (Dehydration)	Group 3 (Forced Impregnation)	Group 4 (Gas Curing)	Cumulative Total Shrinkage (%)
Kidney (Glomerular Area, µm^2^)	4250 ± 210	4110 ± 195	3890 ± 180	3774 ± 165	11.2 ± 1.8%
Testis (Tubule Diameter, µm)	245 ± 12	238 ± 10	200 ± 11	177 ± 9	27.6 ± 3.1%
Ovary (Parenchymal Area, mm^2^)	12.4 ± 0.8	11.9 ± 0.7	10.1 ± 0.6	9.4 ± 0.5	24.2 ± 3.5%
Uterus (Luminal Diameter, µm)	320 ± 18	312 ± 15	288 ± 14	277 ± 12	13.5 ± 2.1%

Note: Stage-dependent dimensional metrics reflect consecutive silicone plastination processing steps from post-fixation baseline to final gas curing.

## Data Availability

The original contributions presented in this study are included in the article. Further inquiries can be directed to the corresponding author.
